# Accuracy of anthropometric measurements by general practitioners in overweight and obese patients

**DOI:** 10.1186/s40608-017-0158-0

**Published:** 2017-06-29

**Authors:** Paul Sebo, François R. Herrmann, Dagmar M. Haller

**Affiliations:** 1 Primary Care Unit, Faculty of Medicine, University of Geneva, Geneva, Switzerland; 2 Department of Internal Medicine, Rehabilitation and Geriatrics, Geneva University Hospitals and Geneva University, Geneva, Switzerland; 3 Department of Community, Primary Care and Emergency Medicine, Geneva University Hospitals, Geneva, Switzerland; 4 Department of Pediatrics, Geneva University Hospitals, Geneva, Switzerland

**Keywords:** Measurement error, Anthropometric measurements, Primary care, Body mass index

## Abstract

**Background:**

We recently showed that abdominal obesity measurements (waist and hip circumference, waist-to-hip ratio) were inaccurate when performed by general practitioners (GPs). We hypothesise that measurement error could be even higher in overweight and obese patients due to difficulty in locating anatomical landmarks. We aimed to estimate GPs’ measurement error of general (weight, height and body mass index (BMI)) and abdominal obesity measurements across BMI subgroups.

**Methods:**

This cross-sectional study involved 26 GPs in Geneva, Switzerland. They were asked to take measurements on 20 volunteers within their practice. Two trained research assistants repeated the measures after the GPs (“gold standard”). The proportion of measurement error was computed by comparing the GPs’ values (*N* = 509) to the average value of two measurements taken in turn by the research assistants and stratified by BMI subgroup (normal/underweight: <25 kg/m^2^, overweight: 25 ≤ BMI < 30 kg/m^2^, obese: ≥30 kg/m^2^).

**Results:**

General obesity measurements were less prone to measurement error than abdominal obesity measurements, regardless of the BMI subgroup. The proportions of error increased across BMI subgroups (except for height), and were particularly high for abdominal obesity measurements in obese patients.

**Conclusions:**

Abdominal obesity measurements are particularly inaccurate when GPs use these measurements to assess overweight and obese patients. These findings add further strength to recommendations for GPs to favour use of general obesity measurements in daily practice, particularly when assessing overweight or obese patients.

## Background

The high prevalence of obesity has become a major global health challenge, since obesity is associated with severe health consequences, contributing to the increase in cardiovascular morbidity and mortality [1, 2]. Early detection by general practitioners (GPs) of patients at high risk for the development of cardiovascular disease is therefore essential. Anthropometric measurements are a non invasive and inexpensive method to assess patients’ nutritional status and have been suggested for wide use in clinical practice. Measuring abdominal in addition to general obesity has been recommended in order to improve cardiometabolic risk assessment, [3, 4] because the pattern of fat distribution has been shown to have a large influence on cardiometabolic risk [5] and, as a consequence, abdominal obesity seems to predict the development of cardiovascular diseases better than overall obesity [6–8].

The waist circumference (WC) and the waist-to-hip ratio (WHR), which is determined by dividing the WC by hip circumference (HC), have become widely accepted measures for assessing abdominal obesity [9, 10]. However, these abdominal obesity anthropometric measurements suffer from higher measurement error than body mass index (BMI), even when they are performed by specially trained measurers [11]. We previously showed in two studies conducted in primary care settings that, in contrast to BMI, obesity was not accurately detected by these abdominal obesity measurement methods and that these measures led to frequent diagnostic misclassification [12, 13]. This could be related to the fact that specific manipulations are required to take these measurements and that GPs are less used to them than they are to measuring BMI.

Measurement error could be particularly high in overweight and obese patients due to difficulty in locating anatomical landmarks [11]. To our knowledge, very few studies examined the influence of BMI subgroup on the risk of measurement error and none in primary care settings. WC and HC were found to have high reliabilities regardless of BMI subgroup in a small study by Wang (*n* = 76), where the participants were measured by a research assistant twice with a 10-min interval [14]. Another small study by Nordhamn (*n* = 51), in which WC and HC were measured by two raters showed that reliabilty tended to decrease in overweight participants for WC and WHR, but not for HC [15]. Note however that these two studies suffer from important limitations: the absence of a gold standard precluded the true assessment of measurement error, and the authors used Asian definitions of BMI subgroups, with lower BMI cut-offs.

Our objective was thus to estimate GPs’ measurement error of general (weight, height and BMI) and abdominal obesity (WC, HC, WHR) anthropometric measurements across BMI subgroups.

## Methods

This is a secondary analysis of data collected in a study of the accuracy of anthropometric measurements in general practice [13]. The methods have been described in detail elsewhere and are briefly summarized here. The research protocol was aproved by the local research ethics committee.

### Recruitment of doctors and volunteers

This study involved a convenience sample of 26 general practitioners (GPs) practicing in the canton of Geneva, Switzerland. The GPs were asked to recruit 20 adult volunteers among their patients, ten for each of two pre-planned measurement sessions. The patients’ next scheduled appointment was synchronized with one of the measurement sessions. In the original study doctors were randomly assigned to two separate groups for the second measurement session. The intervention group received special training in anthropometric measures (the doctors received a training document, prepared by the authors, explaining the appropriate measurement methods according to international recommendations), [16–18] the other acted as control. Since the intervention did not appear to be associated with a significant improvement in GPs’ measurement accuracy, measurements from both groups and both sessions were pooled for the present analysis [13].

### Data collection

The measurement sessions took place within the normal routine of the practice. The GPs were asked to perform the measurements as usual, within the consultation. After having been measured by the GP in his/her consultation room, and while the GP was taking care of the next patient, each volunteer was measured in turn by the two research assistants in a quiet room, close to the consultation room. The research assistants took the measurements according to the recommended procedure for which they had been trained (see below). They were asked to take the measurements with a calibrated flat beam scale for mobile use (SECA 877, scale division: 100 g, capacity: 200 kg), a stadiometer (SECA 217, graduation length: 1 cm, range: 20-205 cm) and a measuring tape.

### Training of the research assistants

Prior to the start of the study, a specialist in anthropometric measurements provided a theoretical and practical training to the two research assistants. As part of this training, a dozen volunteers were measured at the same time by the specialist and the research assistants to confirm that the measurements were accurate. The training was based on international guidelines (see Appendix 1) [16–18]. The instructor of the research assistants was a senior attending physician at Geneva University Hospitals, in the department of therapeutic education for obesity and chronic diseases.

Note that the research assistants were already rather skilled before this training, because they had previously been trained in anthropometric measurements as part of the Bus Santé study (an annual cross-sectional population-based survey collecting data on cardiovascular risk factors in the canton of Geneva).

### Gold standard

The average values of measurements performed by the two trained research assistants was used as a gold standard against which GPs’ measurements were compared to assess the accuracy of GPs’ measurements.

### Statistical analysis

We used frequencies to describe the GPs’ characteristics. We assessed the inter-observer variability between the two research assistants by computing the technical error of measurement (TEM) for each anthropometric measurement. We also computed %TEM (TEM/mean × 100), a measure of the coefficient of variation of TEM, because it is difficult to compare TEMs directly due to the positive association between TEM and measurement size. Then, the difference between the GP’s measurements and the gold standard (=measurement error) was computed for each volunteer. We verified the assumption of normality using the Shapiro-Francia test [19]. One-sample t-tests were then performed to compare the GPs’ and the research assistants’ measurements, and we checked whether mean differences were statistically significantly different from zero. Analyses were stratified by BMI subgroup into underweight/normal (BMI <25 kg/m^2^), overweight (25 ≤ BMI <30 kg/m^2^) and obese participants (BMI ≥30 kg/m^2^). Since only a small subgroup of patients were underweight (*n* = 16, 3.1%) these were grouped with normal participants. Mean relative differences (by dividing the absolute differences by the average values of the measurements undertaken by the research assistants), [13, 20] i.e. the percentage error of the measurements, were preferred to mean absolute differences, because they allowed us to make comparisons between BMI subgroups and anthropometric measurements, as relative values do not depend on the magnitude of these measurements. Then, using oneway analysis of variance (ANOVA), each difference was compared among the three BMI subgroups, which were also compared using Cuzick’s nonparametric test for trend across ordered groups. Finally, multiple linear regression taking into account the repeated measures design, was performed to assess the association of BMI subgroups with the measurement errors after taking into account potential confouders (session (before/after training) and group (intervention/control)). As a measurement difference of <3% is unlikely to be clinically relevant, [11] we decided to consider that the measurements were accurate when the relative measurement errors were <3%.

Statistical significance was set at a two-sided *p*-value ≤0.05. We performed all statistical analyses with STATA version 14.0.

## Results

Participating GPs were aged 44.1 years on average (standard deviation (SD) 6.1, range 33–59), and 58% were women; they were experienced doctors (years since certification 16.3 (SD 5.8), range 7–32). The GPs and two research assistants conducted measurements on 509 volunteers.

The mean differences between the research assistants was very small (for weight: 0.002 kg (SD 0.09); for height: 0.03 cm (SD 0.15); for WC: 0.02 cm (SD 0.14); for HC: 0.01 cm (SD 0.06)). These findings were confirmed by the values of TEMs and %TEMs (for weight: TEM 0.006 kg (%TEM 0.008%); for height: 0.020 cm (0.012%); for WC: 0.015 cm (0.017%); for HC: 0.004 cm (0.004%)).

Based on the measurements made by the research assistants, participants were divided into three BMI subgroups, as follows: BMI <25 kg/m^2^ (normal or underweight: *N* = 237 (47%)), 25 ≤ BMI <30 kg/m^2^ (overweight: *N* = 174 (34%)) and BMI ≥30 kg/m^2^ (obese, *N* = 98 (19%)).

Overall, the mean relative measurement differences, computed from the absolute measurement differences (see Appendix 2), were not associated with BMI subgroups in crude analysis, and were smaller for weight, height, BMI and HC, compared to WC and WHR (Table 1). Height was the most accurate anthropometric measurement (measurement differences: 0.07% (SD 0.58) in underweight/normal, 0.04% (1.00) in overweight and 0.07% (0.72) in obese participants). Only height, BMI and HC were not statistically different when measured by the GPs or the research assistants. For weight, although the differences were small, they were statistically significant (0.36% measurement error for the normal group (absolute difference: 0.22 kg), 0.44% for the overweight group (absolute difference: 0.30 kg) and 0.37% for the obese group (absolute difference: 0.34 kg)).

**Table 1:** Mean relative differences, expressed in percentage (SD), between general practitioners’ anthropometric measurements and the gold standard in normal and underweight (BMI <25 kg/m^2^), in overweight (25 ≤ BMI <30 kg/m^2^) and in obsese (BMI ≥30 kg/m^2^) participants

	Normal/Underweight (*n* = 237)	*p*-value for difference in mean error	Overweight (*n* = 174)	*p*-value for difference in mean error	Obese (*n* = 98)	*p*-value for difference in mean error	*p*-value for difference in mean error among the three groups
Weight [kg]	0.36 (1.34)	<0.001	0.44 (1.21)	<0.001	0.37 (1.19)	0.003	0.81
Height [cm]	0.07 (0.58)	0.06	0.04 (1.00)	0.59	0.07 (0.72)	0.34	0.31
BMI [kg/m^2^]	0.21 (1.78)	0.07	0.49 (2.28)	0.05	0.22 (1.70)	0.21	0.31
Waist circ [cm]	3.17 (5.78)	<0.001	2.26 (4.34)	<0.001	2.07 (5.03)	<0.001	0.10
Hip circ [cm]	0.49 (5.36)	0.16	0.53 (6.07)	0.25	0.32 (4.12)	0.44	0.14
WHR	3.95 (7.82)	<0.001	2.40 (12.84)	0.02	1.92 (6.75)	0.01	0.13

*N* = 509 participants measured by 26 GPs

In multivariable analysis (Table 2), the proportions of error were higher in overweight and obese compared to underweight/normal participants, they increased across BMI subgroups, except for height (ex: for weight, the coefficients were 0 for normal/underweight (baseline), 0.46% for overweight and 0.91% for obese participants), and they increased more for the abdominal compared to the general obesity anthropometric measurements, though the association with BMI subgroups did not follow a trend in a statistically significant manner, except for WHR. In addition, since a measurement difference of <3% is unlikely to be clinically relevant, [11] the differences in proportions of error between overweight/obese and underweight/normal participants were clinically not significant for general obesity measurements. In contrast, they were clinically relevant for WC, HC and WHR.

**Table 2:** Adjusted mean relative differences, compared to baseline BMI subgroup (BMI <25 kg/m^2^), between general practionners’ anthropometric measurements and the gold standard based on multiple linear regression model and compared with a nonparametric test for trend across ordered groups

Characteristics	Adjusted mean relative differences (%)^a^	*p*-value	95% CI	*p*-value for trend
Weight [kg]				
BMI subgroup				0.859
25–29.9 kg/m^2^	0.46	0.002	0.17 to 0.75	
≥ 30 kg/m^2^	0.91	<0.001	0.45 to 1.38	
Height [cm]				
BMI subgroup				0.325
25–29.9 kg/m^2^	0.11	0.16	−0.04 to 0.26	
≥ 30 kg/m^2^	0.01	0.10	−0.18 to 0.18	
BMI [kg/m^2^]				0.605
BMI subgroup				
25–29.9 kg/m^2^	1.23	<0.001	0.71 to 1.74	
≥ 30 kg/m^2^	2.23	<0.001	1.28 to 3.18	
Waist circumference [cm]				0.077
BMI subgroup				
25–29.9 kg/m^2^	3.59	<0.001	2.38 to 4.80	
> =30 kg/m^2^	6.69	<0.001	4.78 to 8.61	
Hip circumference [cm]				0.242
BMI subgroup				
25–29.9 kg/m^2^	2.58	<0.001	1.59 to 3.58	
≥ 30 kg/m^2^	7.37	<0.001	5.85 to 8.90	
Waist-to-hip ratio				0.002
BMI subgroup				
25–29.9 kg/m^2^	5.45	<0.001	3.90 to 6.99	
≥ 30 kg/m^2^	8.20	<0.001	6.30 to 10.11	

*N* = 509 participants measured by 26 GPs

^a^Using multiple linear regression, adjusting for visit (before/after training) and group (intervention/control)

## Discussion

### Summary

Our findings indicate that GPs’ weight, height, BMI and HC measurements are more accurate than their measures of WC and WHR, with errors becoming increasingly more important in higher BMI subgroups.

### Comparison with existing literature

Several authors showed that general obesity measurements were more reliable than abdominal obesity measurements and we recently confirmed these findings when these measurements were taken by GPs within their own practice [12, 21, 22]. As suggested in our previous paper, [13] these results are probably explained by the fact that weight and height measurements are universally known and performed using a relatively simple procedure. In comparison, abdominal obesity measurements are newer concepts and require specific manipulation. We discussed the role of GPs’ knowledge and their usual practice in anthropometrics in our previous paper [13]. We showed that, compared to weight, height and BMI, a majority of GPs hardly ever used the abdominal obesity measures and their knowledge regarding these measurements was relatively low.

We showed statistically significant results regarding the mean relative differences between GPs’ weight measurement and the gold standard. However, these differences are not clinically relevant (0.36% measurement error for normal group (absolute difference: 0.22 kg), 0.44% for overweight group (absolute difference: 0.30 kg) and 0.37% for obese group (absolute difference: 0.34 kg)).

We did not record the gender of the volunteers; therefore, we cannot compute the percentage of misclassification, as the definition of abdominal obesity differs by gender. However, in a previous study, [12] we showed that only 1% of the volunteers were misclassified when the measurements were based on the BMI, compared to 6% when using WC measurement, and 23% when using WHR determination.

Our study suggests that the proportions of error increase across BMI subgroups, except for height. These results slightly contrast with a small study, in which Wang examined the association between BMI subgroup and intrarater reliability. Two unexperimented research assistants received training and together measured WC and HC on 76 participants, twice within a 10-min interval: one was responsible for placing the tape and the other for reading the tape and recording the data. The reliability of these measurements was found to be high for all subgroups, without significant differences across BMI subgroups [14]. The design of this study did not include a gold standard, thus precluding the assessment of measurement error and direct comparison with our findings. In addition, the authors used Asian definitions of overweight and obesity to define BMI subgroups, with lower BMI cut-offs. In another study by Nordhamn [15], WC and HC were measured by two raters in 26 overweight and 25 lean participants. Each participant was measured four times, three times on the first occasion and once on the second. The authors concluded that reliabilty decreased in overweight participants for WC and WHR determination, but not for HC [15]. This study also had important limitations, which makes comparisons with our results difficult: measurement error could not be calculated (no gold standard), WC and HC were measured with the participant in supine position, the definitions of BMI subgroups were unusual and only two groups were included (lean: BMI < 26 kg/m^2^, overweight: BMI ≥ 26 kg/m^2^). To our knowledge no such studies have been carried out in primary care settings.

### Limitations

First, the GPs were not selected at random (convenient sample); as a consequence, our findings may be too conservative, as these GPs may have been more concerned with the subject covered by our study, and therefore may take the anthropometric measurements more frequently and/or more carefully. Second, the study was carried out only in the Geneva area and the study sample may not be representative of all GPs in Switzerland, or Europe more broadly. Third, the study sample was biased towards a higher proportion of overweight (33.2%) and obese (21.6%) volunteers than in the general population in Switzerland [23]. Fourth, we did not record the age and gender of the volunteers, so we cannot provide adjusted mean differences.

### Strengths

The study was undertaken within the normal conditions of day-to-day clinical practice and involved GPs with no particular training in anthropometric measurements. There were only minimal differences between the two research assistants in their mean measurements; this added strength to the value of our gold standard. Misclassification was unlikely, as we used the research assistants’ measurements to stratify the subjects into BMI subgroups. Finally, the BMI subgroups were defined using the usual WHO definitions of overweight and obesity [24].

## Conclusion

Our study suggests that the abdominal obesity measurements are particularly inaccurate when GPs use these to assess overweight and obese patients. We recommend that GPs essentially use general obesity measurements (i.e. weight, height and BMI determination) in daily practice, particularly when assessing overweight or obese patients.

## Appendix 1

### Points emphasised during training of the research assistants (two one-hour sessions)

#### WEIGHT

The participant should remove his shoes and all clothes except underwear, and then step on the centre of the scale, remaining in a relaxed position.

Weight is recorded to the nearest 0.05 kg; if the participant refuses to remove his clothes, 1 kg is substracted from the measurement reading to account for the garments worrn and the refusal is reported.

#### HEIGHT

The participant should remove his shoes and all clothes except underwear, and stand erect on the floorboard of the stadiometer with his back on the side of the vertical board; the weight should be evenly distributed on both feet, the legs closed and stretched, the arms to the sides and the shoulders relaxed; the heels, buttocks and back should slightly touch the vertical board.

The participant is asked to look straight ahead, inhale deeply and stand fully erect while the examiner lowers the horizontal bar to the head crown with hair compressed, and takes the measurement to the nearest 0.1 cm.

#### WAIST CIRCUMFERENCE

The participant should remove his shoes and all clothes except underwear, and stand erect; he is asked to roll up the shirt/sweater and to lower the trouser/skirt waistband, so the examiner can palpate the hip area to identify the measurement reference points, and to mark the level of measurement (the midpoint between the lowest rib and the iliac crest). The measuring tape is placed horizontally, with sufficient tension to avoid slipping off but without compressing the skin.

The measurement is made at the end of a normal expiration, twice to the nearest 0.1 cm, the arms of the participant to the sides; if the difference between the two recorded measurements is greater than 0.5 cm, a third measurement is taken, and the mean of the two nearest values is recorded.

#### HIP CIRCUMFERENCE

The participant should remove his shoes and all clothes except underwear, and stand erect; he is asked to lower the trouser/skirt waistband, so the examiner can palpate the hip area. The tape is placed at the maximum extension of the buttocks, horizontal to the floor, with sufficient tension to avoid slipping off but without compressing the skin.

The measurement is made twice to the nearest 0.1 cm, the arms of the participant to the sides; if the difference between the two recorded measurements is greater than 0.5 cm, a third measurement is taken, and the mean of the two nearest values is recorded.

#### CUT-OFF VALUES

*Body mass index (BMI)*: healthy weight when 18.5 ≤ BMI < 25 kg/m^2^, overweight when 25 ≤ BMI < 30 kg/m^2^, obesity when BMI ≥ 30 kg/m^2^.

*Waist circumference (WC) and waist-to-hip ratio (WHR)*: abdominal obesity in men when WC ≥ 102 cm and/or WHR ≥ 0.95; abdominal obesity in women when WC ≥ 88 cm and/or WHR ≥ 0.8.

## Appendix 2

**Table 3:** Mean absolute differences (SD) between general practitioners’ anthropometric measurements and the gold standard in normal and underweight (BMI <25 kg/m^2^), in overweight (25 ≤ BMI <30 kg/m^2^) and in obsese (BMI ≥30 kg/m^2^) participants. *N* = 509 participants measured by 26 GPs

	Normal/Underweight (*n* = 237)		Overweight(*n* = 174)		Obese(*n* = 98)		Total(*n* = 509)		Oneway ANOVA
Mean absolute difference	Mean	SD	Mean	SD	Mean	SD	Mean	SD	*p* value
Weight [kg]	−0.22	0.82	−0.30	0.91	−0.34	1.15	−0.27	0.92	0.46
Height [cm]	−0.12	0.97	0.07	1.63	−0.11	1.17	−0.05	1.27	0.30
BMI [kg/m^2^]	−0.05	0.40	−0.14	0.62	−0.07	0.57	−0.08	0.52	0.22
Waist circ [cm]	2.41	4.37	2.02	3.94	2.11	5.27	2.22	4.41	0.65
Hip circ [cm]	−0.53	5.05	0.48	6.37	0.29	4.70	−0.03	5.48	0.15
WHR	0.03	0.06	0.02	0.13	0.02	0.06	0.02	0.09	0.36

## Abbreviations

BMI: Body mass index
GPs: General practitioners
HC: Hip circumference
WC: Waist circumference
WHR: Waist-to-hip ratio
